# Further Optimization of the Reliability of the 28-Joint Disease Activity Score in Patients with Early Rheumatoid Arthritis

**DOI:** 10.1371/journal.pone.0100544

**Published:** 2014-06-23

**Authors:** Liseth Siemons, Peter M. ten Klooster, Harald E. Vonkeman, Mart A. F. J. van de Laar, Cees A. W. Glas

**Affiliations:** 1 Arthritis Center Twente, Department of Psychology, Health & Technology, University of Twente, Enschede, the Netherlands; 2 Arthritis Center Twente, Department of Rheumatology, Medisch Spectrum Twente, Enschede, the Netherlands; 3 Department of Research Methodology, Measurement and Data Analysis, University of Twente, Enschede, the Netherlands; University of Texas Southwestern Medical Center, United States of America

## Abstract

**Background:**

The 28-joint Disease Activity Score (DAS28) combines scores on a 28-tender and swollen joint count (TJC28 and SJC28), a patient-reported measure for general health (GH), and an inflammatory marker (either the erythrocyte sedimentation rate [ESR] or the C-reactive protein [CRP]) into a composite measure of disease activity in rheumatoid arthritis (RA). This study examined the reliability of the DAS28 in patients with early RA using principles from generalizability theory and evaluated whether it could be increased by adjusting individual DAS28 component weights.

**Methods:**

Patients were drawn from the DREAM registry and classified into a “fast response” group (N = 466) and “slow response” group (N = 80), depending on their pace of reaching remission. Composite reliabilities of the DAS28-ESR and DAS28-CRP were determined with the individual components' reliability, weights, variances, error variances, correlations and covariances. Weight optimization was performed by minimizing the error variance of the index.

**Results:**

Composite reliabilities of 0.85 and 0.86 were found for the DAS28-ESR and DAS28-CRP, respectively, and were approximately equal across patients groups. Component reliabilities, however, varied widely both within and between sub-groups, ranging from 0.614 for GH (“slow response” group) to 0.912 for ESR (“fast response” group). Weight optimization increased composite reliability even further. In the total and “fast response” groups, this was achieved mostly by decreasing the weight of the TJC28 and GH. In the “slow response” group, though, the weights of the TJC28 and SJC28 were increased, while those of the inflammatory markers and GH were substantially decreased.

**Conclusions:**

The DAS28-ESR and the DAS28-CRP are reliable instruments for assessing disease activity in early RA and reliability can be increased even further by adjusting component weights. Given the low reliability and weightings of the general health component across subgroups it is recommended to explore alternative patient-reported outcome measures for inclusion in the DAS28.

## Introduction

If a concept or condition is too complex to measure with a single instrument, multiple measurements are often combined into a linear composite score (i.e. an index measure). For rheumatoid arthritis (RA) the 28-joint Disease Activity Score (DAS28) is such an index measure, widely used for determining a patient's degree of disease activity [1]. It consists of 4 different individual components: a 28-tender joint count, a 28-swollen joint count, a patient-reported rating of general health, and a non-specific acute phase reactant of systemic inflammation which can be either the erythrocyte sedimentation rate (ESR) or the C-reactive protein (CRP). Each component has its own specific weight in the composite score, based on canonical discriminant functions for classifying high and low disease activity.

The DAS28 has received much attention over the years and has been shown to be a valid measure [1], [2]. However, since reliability is a prerequisite for validity [3], the index should be reliable as well. While several studies have already determined the reliability of the DAS28 using Cronbach's Alpha [2], [4], [5], it is not appropriate to use this internal consistency measure with an index measure, as opposed to scales. Where a scale consists of correlated items which all measure the same construct, an index consists of items which are not necessarily highly correlated but which are considered indicators because they themselves define the construct [6]. As a result, the components might measure completely different aspects of disease activity, which is also the case with the DAS28. This poses significant methodological challenges for reliability testing. To overcome these challenges, generalizability theory [7], [8] can be used to estimate the reliability of an index score by disentangling different sources of error.

As such, the first aim of this study was to determine the reliability of the DAS28 using generalizability theory. Since reliability is a concept defined relative to a specific population of patients [9], the second aim of this study was to examine whether the reliability of the index is acceptably high in relevant subpopulations and whether this reliability can be increased by adjusting the weightings of the individual component scores within the DAS28, which was shown to be the case.

## Methods

### Ethics Statement

As evaluated by the ethics committees of the participating hospitals, and in accordance with Dutch law, no ethical approval was required because data collection took place in daily clinical practice. Nevertheless, informed consent was obtained from each patient.

### Patients

Patients were drawn from the remission induction cohort of the Dutch Rheumatoid Arthritis Monitoring (DREAM) registry [10]. This observational multicenter cohort started in 2006 and, although patient recruitment for this cohort has stopped in 2012, data collection of included patients is still ongoing. For this study, all data available at 0, 3, 6, 9, and 12 months were accessible for analyses. To be eligible for inclusion in the cohort, patients were DMARD and prednisolone naïve, they were allowed to have a maximum symptom duration of 1 year, and they needed to be 18 years or older. Additionally, they were not in remission, as measured with the DAS28. Early RA classification was based on a clinical diagnosis by the rheumatologist. For the present study, patients were classified into 2 groups as identified in the study by Siemons et al. [11]: a “fast response” group of patients quickly reaching remission and a “slow response” group of patients reaching remission at a slower pace. Because reliability calculations are sample dependent slight differences can be expected between the two response groups.

### Measures

The primary measures of interest were the 28-tender joint count (TJC28), the 28-swollen joint count (SJC28), a 100 millimeter visual analogue scale on general health (GH: where 0 = very good and 100 = very bad), the erythrocyte sedimentation rate (ESR), and the C-reactive protein (CRP). Using these variables, the DAS28-ESR and DAS28-CRP were calculated as follows [12]:
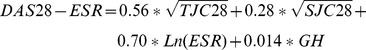
(1)

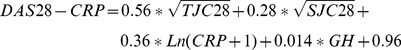
(2)


Patients additionally completed the Health Assessment Questionnaire (HAQ) which measures physical functioning [13] and the 36-item Short Form Health Survey (SF-36) which assesses physical and mental health status [14].

### Statistical analyses

Baseline between-group comparisons were made using independent t-tests for normally distributed continuous variables, Kruskal Wallis tests for variables with skewed distributions, and Chi-Square tests for dichotomous variables.

Although a DAS28 score is assumed to represent a patient's disease activity, in reality this score consists of two parts: 1) the actual (true) score of that patient's disease activity, and 2) random measurement errors [3]. Errors give rise to an under- or overestimation of the true score. This might be due to, among others, certain distractions during test administration, the patient's mood while filling out the test, or a misreading of the items. Reliability is a representation of measurement consistency; it is the ratio between true score variance and observed total score variance, where the latter consists of a true score part and an error part [3]. Higher error variance leads to lower reliability. However, although these basic principles do apply to the individual components of the DAS28, the composite reliability of an index also depends on the interrelationships of its components. Composite reliability is a function of the reliability of the individual components, the weights that are assigned to the components as reflected in the DAS28 formulas, the variances and error variances of the component scores, and the correlations and covariances between the different components. All this can be combined into the following formula [8], [15]:
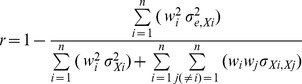
(3)


Where:

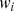
is the weight of component *i*, as defined in the DAS28 formulas 1 and 2 described above;

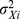
 is the observed variance of component *i*;

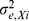
 is the error variance of component *i*, which is a function of the component reliability and the observed variance: 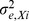  =  (1 - reliability of component i) * 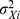;

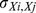
 is the covariance between the two components, which can be rewritten as: *Correlation* 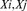
* *Standard deviation* 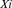
* *Standard deviation* 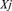.

Note that the nominator of the ratio in formula 3 is the error variance of the index, while the denominator is the total variance.

In this study, the error variances of joint count components were calculated from their observed variance and reliability levels, whereas the error variances of GH and both inflammatory measures were obtained from univariate linear regression analyses. However, two significant problems arose during component reliability computations.

First, it was not appropriate to calculate the reliabilities of the joint count components with Cronbach's Alpha, since Cronbach's Alpha assumes the total score to be a linear combination of all items (i.e. each item is regarded as a parallel test of the other component items) whereas the joint count components are square root transformed in the DAS28. Consequently, split-half reliabilities were determined instead. As demonstrated by Siemons et al. [16], RA is characterized by a definite left-right symmetry of joint involvement. Consequently, the square root sum scores of the left and right joints were considered to represent two parallel tests and their correlation was used as an estimate of reliability.

Second, given the structure of the cohort, with time frames of several weeks or even months between consecutive measurements, it was not possible to perform proper test-retest reliabilities for the single-item components (i.e. ESR, CRP, and GH). Therefore, a generalizability theory principle was used to determine reliability. After running a univariate general linear model analysis on a longitudinal dataset (including all available data at 0, 3, 6, 9, and 12 months) the person variance could be separated from the time variance and reliability could be calculated as the ratio between person variance and total variance.

All computations of reliabilities were performed on the total patient group as well as on the two identified subgroups using SPSS version 21.0.

After calculating the composite reliabilities, it was investigated whether the reliability in both the total as well as the specific patient groups could be optimized by adjusting the component's weights. Optimal weights were computed by minimizing the error variance of the index, that is, the nominator of the ratio in formula 3, subject to the constraint that the total variance of the index does not change. The resulting quadratic optimization problem under quadratic constraints was solved using a procedure developed by Albers, Critchley, and Gower [17]. For more applications, refer to Albers, Critchley, and Gower [18].

## Results

### Patients

A total of 565 patients were included for analysis; 466 from the “fast response” group and 80 from the “slow response” group. Overall, they had a mean age of approximately 58 years, the majority were women (63%) and rheumatoid factor positive (54%), and their baseline disease was characterized by several tender and swollen joints as well as by a diminished physical health status (Table 1).

**Table 1:** Patient characteristics and group comparisons at inclusion.

Variable	Mean (SD) or Median (range)* Total group	Mean (SD) or Median (range)* “Fast response” group	Mean (SD) or Median (range)* “Slow response” group	Significance (*p*) group comparisons
Gender (female)	342/546 (62.6%)	283/466 (60.7%)	59/80 (73.8%)	0.026∼
Age (years)	57.96 (14.31)	57.85 (14.62)	58.59 (12.41)	0.672¶
DAS28-ESR	4.28 (1.45)	4.11 (1.42)	5.22 (1.29)	<0.001¶
DAS28-CRP	4.02 (1.31)	3.88 (1.28)	4.79 (1.24)	<0.001¶
28-Tender joint count	3 (0–28)	3 (0–28)	6 (0–28)	<0.001#
28-Swollen joint count	5 (0–24)	4 (0–24)	6.50 (0–24)	0.001#
GH	44.32 (26.11)	42.52 (25.76)	54.82 (25.83)	<0.001¶
ESR (mm/hour)	21.50 (1–120)	20 (1–111)	30 (7–120)	<0.001#
CRP (mg/l)	9.00 (1–158)	6 (1–158)	11 (1–115)	0.004#
Rheumatoid factor +	274/506 (54.2%)	233/431 (54.1%)	41/75 (54.7%)	0.923∼
SF36 – physical health	37.17 (9.09)	37.93 (9.09)	32.91 (7.88)	0.001¶
SF36 – mental health	48.16 (11.67)	48.55 (11.37)	45.99 (13.07)	0.094¶
HAQ	1.00 (0.72)	0.92 (0.70)	1.41 (0.66)	0.001¶

*The values for gender and rheumatoid factor positivity are the number of patients/number of patients assessed (%).

# Group comparisons performed with Kruskall Wallis tests.

∼ Group comparisons performed with Chi-Square tests.

¶ Group comparisons performed with independent t-tests.

DAS28  =  disease activity score for 28 joints, GH  =  general health, ESR  =  erythrocyte sedimentation rate, CRP  =  C-Reactive Protein, SF36  =  Short Form Health Survey with 36 items, HAQ  =  Health Assessment Questionnaire.

No significant differences were found in age, mental health, or rheumatoid factor positivity between the fast and slow response group, but the slow response group did contain significantly more women. Additionally, the slow response group had a significantly worse disease condition, characterized by higher DAS28 scores, a poorer state of general and physical health, higher levels of inflammatory markers, and a higher number of tender and swollen joints, as compared to the fast response group.

### Reliability calculations

Tables 2–4 show the variances, error variances and reliabilities of the individual components together with their correlations and covariances. All correlations were small to moderate, as is not unusual in index measures. Component reliabilities varied widely both within and between sub-groups, ranging from 0.614 for GH in the “slow response” group to 0.912 for ESR in the “fast response” group. Furthermore, ESR component reliabilities were highest and the TJC28 outperformed the SJC28 across all groups.

**Table 2:** Variances, error variances and reliabilities of the DAS-28 components.

	Total patient group (N = 546)			“Fast response” group (N = 466)			“Slow response” group (N = 80)		
Component	Variance	Error variance	Component Reliability	Variance	Error variance	Component Reliability	Variance	Error variance	Component Reliability
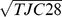	1.756	0.400	0.772	1.580	0.409	0.741	2.085	0.325	0.844
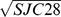	1.521	0.420	0.724	1.522	0.425	0.721	1.342	0.394	0.706
**GH**	681.624	269.687	0.783	663.439	247.769	0.766	666.917	359.309	0.614
**LN(ESR)**	0.779	0.193	0.913	0.807	0.191	0.912	0.477	0.191	0.878
**LN(CRP)**	0.909	0.246	0.805	0.920	0.231	0.806	0.771	0.335	0.773

TJC28  =  28-tender joint count, SJC28  =  28-swollen joint count, GH  =  general health, ESR  =  erythrocyte sedimentation rate,

CRP  =  C-Reactive Protein.

**Table 3:** Pearson's correlations and covariances of DAS-28 components in total patient sample.

	Covariance	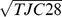	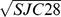	GH	LN(ESR)	LN(CRP)
Pearson's Correlation						
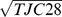		----	0.909	14.818	0.152	0.207
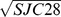		0.555**	----	8.467	0.337	0.402
**GH**		0.427**	0.262**	----	4.129	5.181
**LN(ESR)**		0.131**	0.309**	0.179**	----	0.488
**LN(CRP)**		0.164**	0.343**	0.207**	0.581**	----

**p<0.01, TJC28  =  28-tender joint count, SJC28  = 28-swollen joint count, GH  =  general health, ESR  =  erythrocyte sedimentation rate, CRP  =  C-Reactive Protein.

**Table 4:** Pearson's correlations of the DAS-28 components in the “fast response” group and “slow response” group.

	Pearson's correlation group 2 (N = 80)	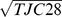	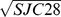	GH	LN(ESR)	LN(CRP)
Pearson's correlation group 1 (N = 466)						
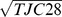		1	0.547**	0.313**	−0.09	−0.032
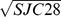		0.545**	1	0.288*	0.143	0.263*
**GH**		0.423**	0.239**	1	0.067	0.104
**LN(ESR)**		0.125**	0.312**	0.166**	1	0.578**
**LN(CRP)**		0.171**	0.342**	0.205**	0.574**	1

**p<0.01, TJC28  = 28-tender joint count, SJC28  = 28-swollen joint count, GH  =  general health, ESR  =  erythrocyte sedimentation rate, CRP  =  C-Reactive Protein.

Using formula 3 resulted in overall reliabilities of 0.85 and 0.86 for the DAS28-ESR and DAS28-CRP composites, respectively. Sub-analyses showed DAS28-ESR reliabilities of 0.85 and 0.82 and DAS28-CRP reliabilities of 0.85 and 0.84 for the “fast response” and “slow response” group, respectively. These results demonstrate that both DAS28 scores are approximately equally reliable across patient groups, that is, the differences were only small and all reliability levels were high (>0.80).

### Optimizing reliability

The results of the optimization of composite reliability by adjusting the weights are shown in Table 5. The original weights are given in the third column. The next three columns give the estimated optimal weights in the total group and in the “fast response” and “slow response” groups, respectively. Further, in the rows labeled “Reliability”, the reliabilities using the original weights and the optimal weights are given.

**Table 5:** Reliabilities of the index measures and optimal weights in subpopulations.

Index measure	Component *	Original Weight	Total patient group (N = 546)	“Fast response” group (N = 466)	“Slow response” group (N = 80)
**DAS28-ESR**	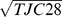	0.560 (0.742)	0.226 (0.299)	0.156 (0.196)	0.739 (1.067)
	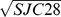	0.280 (0.345)	0.271 (0.334)	0.226 (0.279)	0.355 (0.411)
	**GH**	0.014 (0.366)	0.004 (0.104)	0.004 (0.103)	0.004 (0.103)
	**LN(ESR)**	0.700 (0.618)	0.663 (0.585)	0.650 (0.584)	0.052 (0.036)
**Reliability**	**Original Weights**		0.854	0.848	0.821
	**Optimal Weights**		0.933	0.942	0.859
**DAS28-CRP**	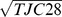	0.560 (0.742)	0.374 (0.496)	0.247 (0.310)	0.720 (1.040)
	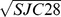	0.280 (0.345)	0.378 (0.466)	0.298 (0.368)	0.356 (0.412)
	**GH**	0.014 (0.366)	0.006 (0.157)	0.005 (0.129)	0.004 (0.103)
	**LN(CRP)**	0.360 (0.343)	0.522 (0.498)	0.571 (0.548)	0.028 (0.025)
**Reliability**	**Original Weights**		0.858	0.852	0.845
	**Optimal Weights**		0.888	0.911	0.858

The weights between brackets are the standardized values by fixing the component variances at 1.

*TJC28  = 28-tender joint count, SJC28  =  28-swollen joint count, GH  =  general health, ESR  =  erythrocyte sedimentation rate, CRP  =  C-Reactive Protein.

In all groups, reliabilities increased after weight optimization. The largest gains were obtained in the total and “fast response” groups by decreasing the weight of the TJC and GH. In the smaller slow response group, on the other hand, the weights of the TJC28 and SJC28 were increased, while the weights of the inflammatory markers and GH were substantially decreased.

## Discussion

Overall, composite reliability levels of 0.85 and 0.86 were found for the DAS28-ESR and DAS28-CRP, respectively. This is sufficiently high for group use and around common thresholds considered sufficient for individual use [19], [20], justifying the use of the DAS28 in both clinical research and clinical practice. Moreover, reliability could be increased even further by optimizing the component weights.

Several findings are worth mentioning when comparing the individual component reliabilities or when optimizing the component weights. At first, it can be observed that ESR had the highest reliability of all DAS28 components. The finding that this measure of inflammation was also more reliable than the CRP measure might be explained by their different responsiveness to changes in inflammatory stimuli. While the ESR is a relatively stable measure over time, which responds slowly to changes and reflects the disease activity of the past few weeks, the CRP fluctuates more heavily due to a more rapid response to short-term changes in the inflammatory stimuli [12], [21], [22]. The ESR is also given a higher weighting than the CRP in the DAS28 formulas and that remained to be the case after weight optimization.

When looking at the joint count reliabilities, the swollen joint count had a lower reliability than the tender joint count, consistent with findings from previous studies [9], [23], [24]. Joint counts are sometimes referred to as a semi-objective clinical measures [1] and, as discussed by Pincus [25], they have been shown to be poorly reproducible. Large intra- and interobserver variability is commonly found especially in the swollen joint count [24]. This might be explained by a higher dependency of the swollen joint assessment on factors like the assessors' levels of training and experience, a lack of standardization in examination methods, unclear definitions of swelling, or the degree of joint deformity [24], [25], [26]. After weight optimization, however, the weight of the tender joint count was substantially lowered, even below the weight of the swollen joint count. This corresponds with the common clinical perspective of disease activity in RA. Joint swelling is usually considered to be a more representative measure of inflammation than joint tenderness [27] and has been shown to play a major role in the physician's assessment of disease activity [28].

Finally, it can be observed that the patient reported degree of GH in the “slow response” group had the lowest reliability of all components, even below the recommended reliability threshold for group use (*r*>0.70). Its weight was also substantially decreased after weight optimization. This could be a confirmation of the weakness of this component, as the inclusion of GH in the DAS28 has been often criticized. For instance, previous studies have shown elevated GH scores while none of the other DAS28 components showed any sign of an active disease, possibly due to effects beyond the clinical inflammatory processes of RA [29]. Also, GH ratings have been shown to be different across patients with similar DAS28 scores, dependent on the moment of administration, possibly caused by a response shift [30]. The GH component is also the most subjective component of the DAS28 and, therefore, more susceptible to measurement error. Although it could be argued to solely include the more objective clinical measures, the inclusion of a patient-driven component is desirable given the increased awareness of the importance of the patient perspective in assessing disease activity since the 1980s [31] as reflected by their inclusion in the provisional ACR/EULAR definition of remission in RA [32] as well as in the preliminary core set of disease activity measures [33]. Disease activity in RA is a multifactorial concept and appears to be best measured by both objective clinical measures and patient-reported outcomes as they each address a different aspect of disease activity [27]. Therefore, it would be interesting to explore other, more reliable, patient-reported outcome measures for inclusion in the DAS28. What measure would be best warrants further research. Measures of pain or fatigue appear to be promising alternatives, given the recognition of pain as one of the most important determinants of a patient's global assessment of disease activity [34], [35], [36] and the recommendation to measure fatigue in addition to the other core set measures of RA [37]. But, of course, other patient-reported measures can also be explored.

A possible limitation of the current study is the difference in sample size between the fast response and slow response group. Though this might cause the results of the “fast response” group to be more robust than the results of the “slow response” group, the importance of the tender joint count in the slow response group is consistent with the general belief that approximately 10-20% of patients with RA have secondary fibromyalgia (FM) [38], [39]. FM patients tend to have a lower pain threshold [38], [39] which might explain the higher relevance of the TJC28 rating in this patient group, increasing our confidence in the robustness of the results.

## Conclusions

The DAS28-ESR and the DAS28-CRP are both reliable instruments for assessing disease activity in early RA although reliability can be increased even further by adjusting the individual component weights. Overall, the findings suggest that the largest gains in reliability can be achieved by substantially lowering the weights of the tender joint count and patient-reported general health. Future studies should explore the possibilities of including a better indicator of the patient perspective in the disease activity score.
